# Video consultations for dentistry in primary health care: a pilot study

**DOI:** 10.3389/froh.2026.1829036

**Published:** 2026-05-25

**Authors:** Mercedes Perez-Heredia, Adrián Aparicio-Mota, Diana Jiménez-Rodríguez

**Affiliations:** 1 Distrito Sanitario Poniente de Almeria, Servicio Andaluz de Salud, Almería, Spain; 2 Foundation for Biomedical Research in Eastern Andalusia (FIBAO), Almería, Spain; 3 Department of Nursing, Physiotherapy and Medicine, University of Almería, Almería, Spain

**Keywords:** digital divide, primary health care, rural health services, smartphone, teledentistry, video consultation

## Abstract

**Background:**

Teledentistry is key to achieving equitable healthcare, especially in rural areas. According to the Global Strategy for Oral Health, the use of digital technologies such as video consultations (VCs) makes it possible to overcome geographical barriers, facilitating access to basic services and early detection of diseases.

**Aim:**

To analyse the implementation of video consultations for oral health and patient satisfaction level at Primary Health Care in rural areas.

**Method:**

Descriptive study in adult patients in rural areas of Andalusia (Spain). The intervention was carried out using virtualisation platforms and systems integrated within the Andalusian Public Health System of the Andalusian Health Service (SSPA), ensuring confidentiality. Professionals received prior training through clinical simulation to ensure adequate and humanised performance in remote care.

**Results:**

The use of VCs in primary care showed high effectiveness and overall satisfaction, with 78.6% of consultations being finalised and a high level of patient satisfaction (9). Most patients only required one video consultation (VC) (76.8%). However, significant digital divides were identified, with results showing significant differences between connection groups (*p*-value < 0.001), with those over 55 being the most affected group, as well as 90% of those who needed external help to connect and 50% of those who had connection problems.

**Conclusion:**

Video consultations in dentistry are a valuable tool for rural primary care, improving accessibility and efficiency. Social support is a key to overcome technological difficulties which face older user.

## 1 Introduction

The WHO (World Health Organisation) defines oral health as “the condition of the mouth, teeth and orofacial structures that enables people to perform basic functions such as eating, breathing and speaking, and affects psychosocial dimensions such as self-confidence, well-being and the ability to socialise and work without pain, discomfort or embarrassment” (1). Oral health varies throughout the life, from early childhood to old age, 28 and it is considered an integral part of overall health and helps people participate in society and reach their potential (1).

The Global Strategy on Oral Health 2023–2030, supported by the global agenda, seeks to improve oral health care and access worldwide. One of its fundamental pillars is the optimisation of digital technologies, an approach that is particularly relevant for addressing geographical disparities (2).

For providing equity in a healthy society, it is essential to ensure that everyone has equal access to quality healthcare. This means not only that services are available, but also that they are affordable, acceptable and tailored to each individual's needs, removing geographical, economic, cultural and social barriers (1).

A resilient healthcare system is one that is capable of anticipating, absorbing, adapting and transforming in the face of any type of impact, whether it be health crises, natural disasters or demographic changes. In this context, primary care plays a crucial role. It is the gateway to the health system and the level of care closest to the public (3, 4). In this sense, digital technology offers powerful tools for addressing inequalities in access to and quality of healthcare when programmes are designed to focus on the specific needs of the target audience. The implementation of telemedicine acts as a catalyst for healthcare equity, as remote consultations actively help to overcome the geographical and mobility barriers that traditionally affect rural populations (5). Where a lack of professionals and the remoteness of health centres are significant barriers, tele-dentistry is emerging as a vital solution (6). This technology allows digital access and consultations to be integrated into primary care platforms, making it easier for patients in remote areas to receive guidance, initial diagnoses and follow-up without having to travel. According to the WHO, digital technologies can be used to train local health workers, educate the population on oral hygiene, and early detection of diseases (1). This brings oral care closer to communities that would otherwise be underserved. In this way, teledentistry is not only a technological tool, but also, a mean to achieve Global Target 4.1: to ensure that, by 2030, 80% of countries offer oral care services in a comprehensive manner in their primary care centres, ensuring that no one is left behind (2). In addition, it can provide rapid access to a safe course of action in crowded hospital waiting rooms or dental offices, But as with any health management protocol, its effectiveness and sustainability depend on its proper integration into health services with full confidentiality of patient data (7, 8).

Within telemedicine or tele-dentistry, and due to the need to develop and implement new tools that enable medical care in other conditions and clinical situations, video consultations have emerged with the idea of guaranteeing the quality of healthcare that other services are not providing (9, 10). Currently, studies on video consultations in dentistry are scarce (10–13), and none of them evaluate video consultations in an integrated and routine manner within the scope of public primary care in rural areas. This could become an emerging opportunity for people with chronic conditions and a tool that reduces physical barriers and promotes healthy ageing (14).

As dentistry was one of the specialities that suffered most during COVID-19, mainly due to the formation of aerosols and secretions (15, 16), it became essential to take additional precautions to mitigate this. Unfortunately, guidelines for providing dental treatment during COVID-19 varied around the world, and dental practice was adjusted to regional regulations (17). However, the need to find solutions that could offer patients basic medical care and professionals safety and the re-establishment of their daily practice led to the resumption and improvement of adaptive systems such as teledentistry, which combined digital technology with clinical practice (18–20).

Making the most of the clinical benefits offered by telemedicine depends fundamentally on both patients and healthcare professionals accepting and actively using its applications. Therefore, in addition to researching the effectiveness of video consultations, it is crucial to conduct studies that explore satisfaction, perceived usefulness, and actual use of the various services provided by this technology (10, 11).

The aim of this study was to analyse the implementation of video consultations for oral health and patient satisfaction level at Primary Health Care in rural areas.

## 2 Methodology

This study was conducted in Primary Health Care within the Andalusian Public Health System once the protocol was approved by the Almería Provincial Research Ethics Committee CEI/CEIm code 126/2021 (Andalusia-Spain). Written informed consent was obtained from each patient for participation in the study. The study period ran from 1 May to 14 July 2025, during which time participants were recruited and the video consultations were conducted.

This study was reported in accordance with the Strengthening the Reporting of Observational Studies in Epidemiology (STROBE) statement for observational research (21). It was designed as a descriptive observational study comprising a quantitative assessment of video consultations and patient satisfaction level. Patients’ overall satisfaction with the care received via video consultation was assessed, with a score of 0 indicating no satisfaction and a score of 10 indicating high satisfaction.

No formal power-based sample size calculation was performed, as this was a pilot implementation study with the descriptive aim of assessing the feasibility and operation of video consultations. Sixty consecutive patients were included by convenience sampling within the study period, which allows key proportions to be estimated with reasonable precision (95% CI ≈ ± 13% in the worst case and ≈ ± 10%–11% for proportions close to 0.80). This sample size is a limitation but provides a basis for subsequent studies.

### 2.1 Design

Descriptive study to evaluate the implementation of video consultations in primary care. The study sample consisted of adult patients seen at primary care dental clinics through video consultations. The objective was to obtain information on the impact of implementing care through video consultations using a humanised care guide described by Diana Jiménez (2020) (22). The data collection instrument was developed using the Delphi method (23) with a meeting of experts that included health centre professionals, professionals trained in telemedicine, and dentists. This questionnaire was designed as a self-administered *ad hoc* questionnaire, developed specifically for this study by the working team to evaluate the video consultation.

**Figure 1:**
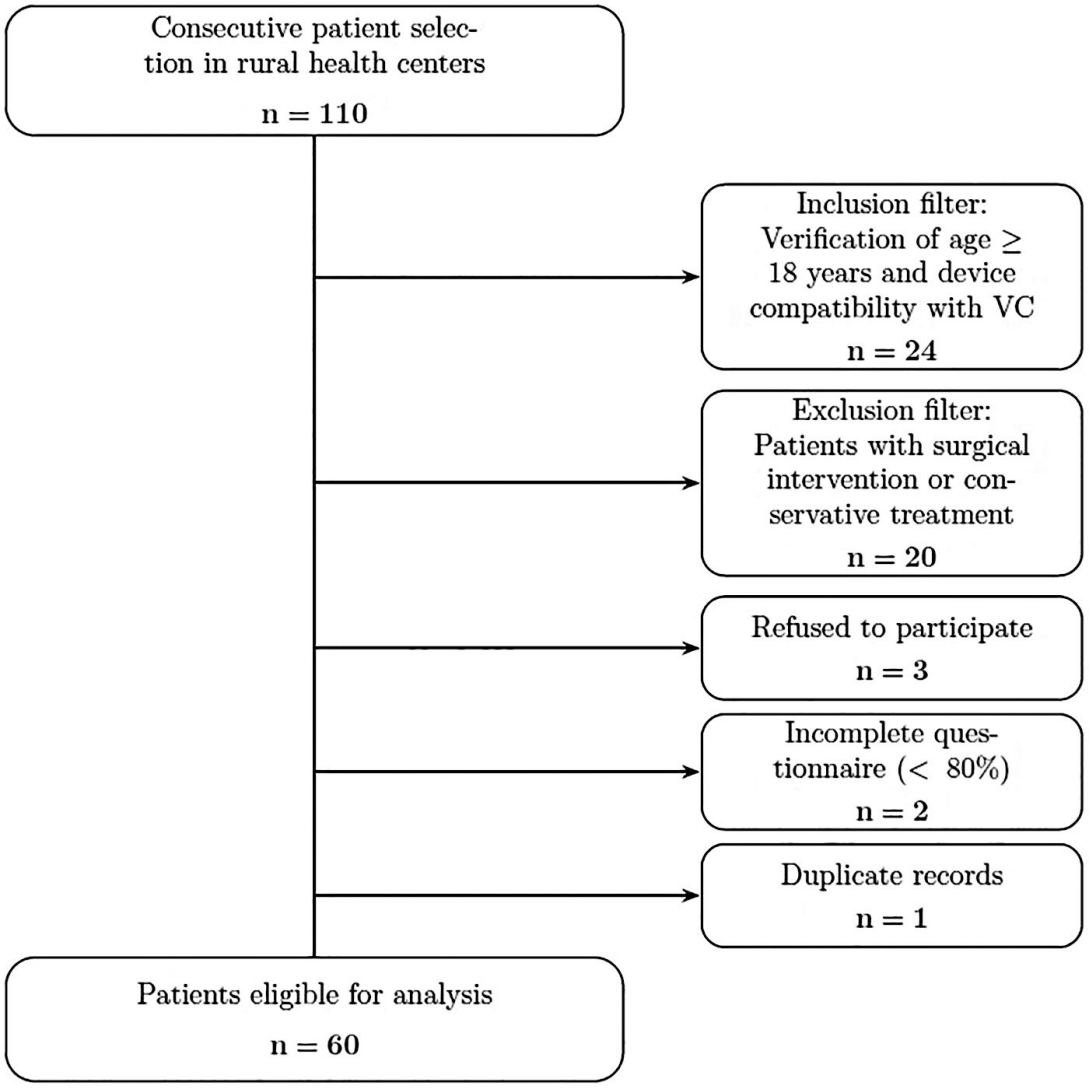
Flowchart of patient selection.

The study population consisted of primary care users over the age of 18 belonging to the basic health zones attached to the Poniente Health District of Almería, which is part of the Public Health Service of Andalusia, Spain, and who had access to a device for video consultations: smartphone or laptop/desktop computer with a camera and microphone, and who voluntarily agreed to participate in the study. Patients who were unable to cooperate or understand the study instructions, and patients requiring surgical or conservative treatment, were excluded. Patients were included consecutively during the study period, provided they met the following criteria; video consultations were offered as part of standard clinical practice, and their use was determined on the basis of clinical appropriateness, i.e., by assessing whether the condition could reasonably be managed through this type of consultation. The patient recruitment flowchart is shown below (Figure 1).

The professional who conducted the video consultations was previously trained in the use of video consultations, both in relation to the management of technological resources and the proper management of the consultation using high-fidelity clinical simulation methodology, specifically in the form of simulated video consultations (24) with the aim of improving professional performance training, following a guide to humanised action in video consultations (25). Intervention

In order to implement video consultations in primary care and ensure the transfer of sensitive data through networks and in accordance with these basic principles, Citrix Virtual Desktops^TM^, Unify Circuit platforms, health information systems such as Diraya, and applications such as Mercurio and Capptura, file-sharing applications, were used, integrated within the Andalusian Public Health System, which guaranteed confidentiality, authenticity, and integrity that other more popular platforms or applications do not have and which are not ideal for this use as they do not meet most of these requirements. These are platforms or applications developed for clinical management in secure environments, guaranteeing data protection and direct integration into information systems and digital health records. Furthermore, the healthcare centres where the video consultations were carried out had the necessary technical equipment to provide this services.

### 2.2 Data analysis

A descriptive analysis of the sociodemographic characteristics of the patients and the clinical variables was performed. For quantitative variables, the data were expressed as measures of central tendency and dispersion, using the arithmetic mean accompanied by its standard deviation (SD) when the variables followed a normal distribution, and the median with the interquartile range (IQR) in cases of non-normal distributions. Additionally, 95% confidence intervals (95% CI) were calculated: exact binomial intervals using the Clopper–Pearson method for qualitative variables, and confidence intervals for the mean based on the Student's t distribution for quantitative variables.

A bivariate analysis was performed to determine whether there were significant differences between sociodemographic variables such as age groups and minutes of video consultation (VC) or problem resolution in the same (final consultation). For quantitative variables, Student's t-test or ANOVA for independent samples was applied in normal distributions, and the non-parametric Mann–Whitney U test or Kruskall Wallis test was applied when distributions were not normal. For the comparison of qualitative variables, the Chi-square test and Fisher's test were used, depending on the expected size of the frequencies in the contingency tables.

Statistical analyses were performed using R Statistical Software (version 4.1.2; R Core Team 2021) and SPSS version 26 (IBM Inc., Armonk, NY, USA). Graphical visualisation was performed using the “ggstatsplot” library (26) implemented in R.

## 3 Results

The variables analysed were age, age groups G1 (18–30), G2 (31–55), G3 > 55, gender, minutes of video consultation (VC), final consultation (resolution of the reason for consultation in the VCs), number of video consultations performed by the patient, VC connection problems and reason for consultation.

The results describing the different variables analysed are shown in Table 1. It can be seen that the most represented age group was G2 (31–55 years) with 56.67% (34 cases), followed by group G3 > 55 years (25.00%, 15 cases) and, finally, G1 (18–30 years) (18.33%, 11 cases). There was a slight predominance of women (53.33%, 32 cases) over men (46.67%, 28 cases).

**Table 1:** Sociodemographic characteristics and characteristics of the VC process.

Variable	*N* = 60/Estimate
Age groups	
G1. 18–30	11 (18.33%) [9.52–30.44]
G2. 31–55	34 (56.67%) [43.24–69.41]
G3. >55	15 (25.00%) [14.72–37.86]
Gender	
Female	32 (53.33%) [40.00–66.33]
Male	28 (46.67%) [33.67–60.00]
Reason for consultation resolved (valid *n* = 55)	
Yes	43 (78.18%) [64.99–88.19]
No	12 (21.82%) [11.81–35.01]
Code reason for consultation	
1 Toothache, neuralgia	12 (20.00%) [10.78–32.33]
10 Tooth fracture	1 (1.67%) [0.04–8.94]
2 Temporomandibular joint dysfunction	6 (10.00%) [3.76–20.51]
3 Results of complementary tests	14 (23.33%) [13.38–36.04]
4 Referral to hospital or other specialists	5 (8.33%) [2.76–18.39]
5 Preoperative	5 (8.33%) [2.76–18.39]
6 Treatment reviews/chronic diseases	9 (15.00%) [7.10–26.57]
7 Infections	3 (5.00%) [1.04–13.92]
8 Second opinion	1 (1.67%) [0.04–8.94]
9 Burning mouth syndrome	4 (6.67%) [1.85–16.20]
Number of VC/PAC (valid *n* = 55)	
1	42 (76.36%) [63.00–86.79]
2	13 (23.64%) [13.21–37.00]
Connection problems (valid *n* = 54)	
No	39 (72.22%) [58.36–83.54]
Connects with external assistance	10 (18.52%) [9.25–31.43]
Yes	5 (9.26%) [3.08–20.30]
VC satisfaction (valid *n* = 33)	8.85 ± 0.87 [8.54–9.16]

Quantitative variables are shown as mean ± SD [95% CI]. Qualitative variables are shown as *n* (%) [95% exact binomial CI]. Percentages were calculated using valid cases for each variable.

Regarding the effectiveness and reason for consultation, the results show that most consultations were finalised [the reason for consultation was resolved through VC in 43 (78.18%) cases]. The most common reasons for consultation were 3: results of complementary tests (23.33%), 1: odontalgia and neuralgia (20.00%) and 6: reviews of treatments or chronic diseases (15.00%).

In terms of frequency and connectivity, most patients had a single VC, 42 (76.36%). Regarding connection problems, most patients had no problems connecting to the VCs (72.22%, 39 cases), 18.52% (10 cases) were able to connect but with external help (family member, carer) and 9.26% (5 cases) reported connection problems.

The level of patient satisfaction was high, with scores between 7 and 10, with a score of 9 (42.42%, 14 cases) being the most frequent.

The results of the analysis comparing the characteristics of the video consultation and the patients based on connection difficulties are shown in Table 2.

**Table 2:** Comparison by connection problems.

Variable	Category	No	Connects with external help	Yes	*p*
VC minutes		8.56 ± 1.85 [7.97–9.16]	10.60 ± 1.35 [9.63–11.57]	10.20 ± 1.92 [7.81–12.59]	<0.01
Number of VC/PAC		1.33 ± 0.48 [1.18–1.49]	1.00 ± 0.00 [1.00–1.00]	1.00 ± 0.00 [1.00–1.00]	0.04
Satisfaction		8.88 ± 0.88 [8.52–9.24]	9.17 ± 0.41 [8.74–9.60]	7.50 ± 0.71 [1.15–13.85]	0.09
Gender	Male	21 (53.85%) [37.18–69.91]	2 (20.00%) [2.52–55.61]	3 (60.00%) [14.66–94.73]	0.15
	Female	18 (46.15%) [30.09–62.82]	8 (80.00%) [44.39–97.48]	2 (40.00%) [5.27–85.34]	
Age group	18–30	10 (25.64%) [13.04–42.13]	1 (10.00%) [0.25–44.50]	0 (0.00%)	<0.01
	31–55	26 (66.67%) [49.78–80.91]	0 (0.00%)	2 (40.00%) [5.27–85.34]	
	>55	3 (7.69%) [1.62–20.87]	9 (90.00%) [55.50–99.75]	3 (60.00%) [14.66–94.73]	
Reason for consultation resolved	No	9 (23.08%) [11.13–39.33]	3 (30.00%) [6.67–65.25]	0 (0.00%)	0.45
	Yes	30 (76.92%) [60.67–88.87]	7 (70.00%) [34.75–93.33]	5 (100.00%) [47.82–100.00]	

Quantitative variables: mean ± SD [95% CI] (Kruskal–Wallis). Qualitative variables: *n* (%) [95% exact binomial CI] (Chi-square/Fisher as appropriate).

Analysis of quantitative variables by connection problem groups showed statistically significant differences in the duration of VC and the number of VCs per patient (VC/PAC). There were also significant differences in the duration of the VC (*p*-value=0.003), with patients who connected with external assistance having a longer average time (10.60 ± 1.35 min) and those who had no problems having a shorter time (8.56 ± 1.85 min). Significant differences were observed in the number of VC/patient (*p*-value=0.032), with patients who had fewer connection problems having more VC (1.33 ± 0.48).

Patient satisfaction was lower in the group that had connection problems (7.50 ± 0.71), although there were no statistically significant differences (*p*-value=0.094).

**Figure 2:**
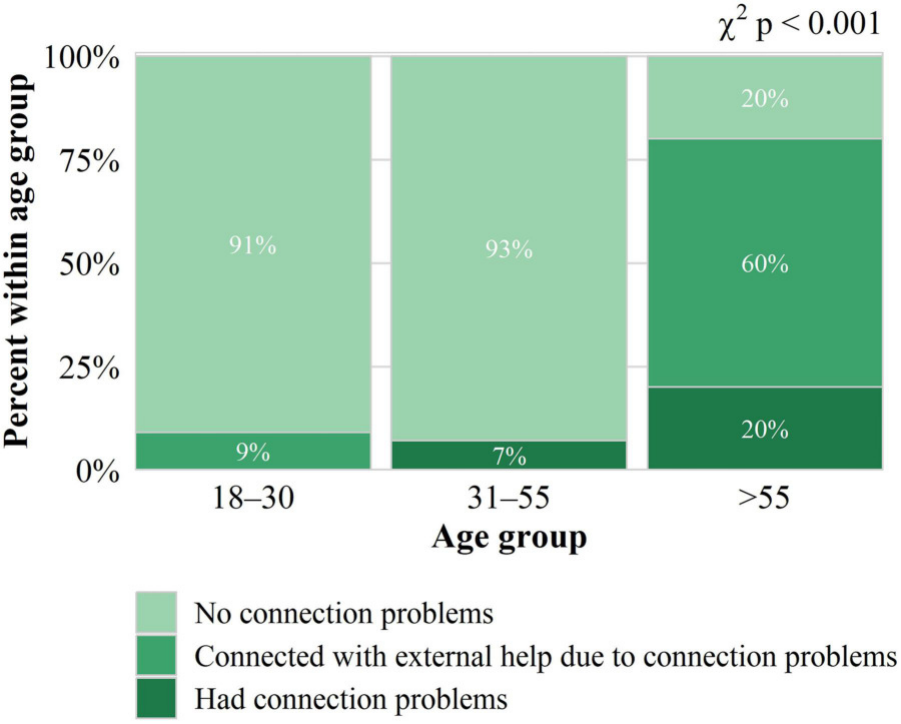
Connection problems by age group.

With regard to the comparison by age groups, the results showed significant differences between the connection groups (*p*-value < 0.001), with G3 (>55 years) associated with connection difficulties, representing 90% of those who connected with external help and 60% of those who reported connection problems (Table 2 and Figure 2).

With regard to gender, the results showed no statistically significant difference between the groups (*p*-value=0.151). However, the group that connected with external help is composed mainly of women (80.0%).

As for the final consultation, no statistically significant differences were found in the resolution of the reason for consultation based on connection problems (*p*-value=0.388), but it should be noted that 100% of participants who had connection problems stated that the reason for the consultation was resolved.

Table 3 shows the comparative analysis of the characteristics of the video consultation and the associated results, stratified by three age groups: G1, G2, and G3. In this regard, the results revealed that there are only statistically significant differences in the number of VCs per patient (*p*-value=0.047). Patients over 55 years of age consistently had a single VC (1.00 ± 0.00), while the younger groups (18–30 years and 31–55 years) had a slightly higher average than one (1.36 ± 0.50 and 1.31 ± 0.47, respectively), suggesting a higher probability of follow-up VCs in these groups.

**Table 3:** Comparison by age group.

Variable	Category	18–30	31–55	>55	*p*
VC minutes		8.27 ± 1.49 [7.27–9.27]	10.28 ± 4.32 [8.72–11.84]	10.00 ± 1.89 [8.95–11.05]	0.09
Number of VC/PAC		1.36 ± 0.50 [1.02–1.70]	1.31 ± 0.47 [1.13–1.49]	1.00 ± 0.00 [1.00–1.00]	0.04
Satisfaction		9.33 ± 0.87 [8.67–10.00]	8.80 ± 0.86 [8.32–9.28]	8.44 ± 0.73 [7.89–9.00]	0.09
Gender	Male	7 (63.64%) [30.79–89.07]	15 (44.12%) [27.19–62.11]	6 (40.00%) [16.34–67.71]	0.44
	Female	4 (36.36%) [10.93–69.21]	19 (55.88%) [37.89–72.81]	9 (60.00%) [32.29–83.66]	
Reason for consultation resolved	No	0 (0.00%)	8 (27.59%) [12.73–47.24]	4 (26.67%) [7.79–55.10]	0.17
	Yes	11 (100.00%) [71.51–100.00]	21 (72.41%) [52.76–87.27]	11 (73.33%) [44.90–92.21]	

Quantitative variables: mean ± SD [95% CI] (Kruskal–Wallis). Qualitative variables: *n* (%) [95% exact binomial CI] (Chi-square/Fisher as appropriate).

Mean satisfaction was highest in the 18–30 age group (9.33 ± 0.87) and decreased progressively with age, being lowest in the >55 age group (8.44 ± 0.73) (*p*-value=0.094).

The age groups were homogeneous in terms of gender (*p*-value=0.443) and resolution of the reason for consultation (*p*-value=0.139). It should be noted that in the youngest group (18–30 years), 100.0% of cases were resolved, unlike the 31–55 year old group (72.41%) and the >55 year old group (73.33%), where a quarter of patients reported that their reason for consultation had not been resolved.

## 4 Discussion

More than 3.5 billion people worldwide are affected by oral diseases (27). This demonstrates the urgent need to find more accessible and affordable ways to receive dental care. According to the World Health Organisation (WHO), the definition of telehealth involves providing clinical support to patients through the use of various types of information and communication technologies (ICT) with the aim of improving health outcomes (28, 29). Choosing the right tools for this study was essential to ensure the security of sensitive data. Therefore, a set of platforms and software integrated into the Andalusian Public Health System was chosen. The solutions used, such as CitrixTM virtualisation software, Circuit platforms, the Diraya Health Information System, and the Mercurio and Capptura applications, ensure the principles of confidentiality, authenticity, and integrity (30, 31). In addition, the American Dental Association (ADA) recognises that teledentistry is a valuable tool for professionals to reach more patients. However, it also emphasises that virtual care must be of the same quality as that offered in person. This poses a challenge, as dentistry is a manual practice and many professionals find it difficult to understand how a remote examination can be as thorough and accurate as an in-person one (32). Despite the boom in tele-dentistry research following COVID-19 and evidence of its effectiveness (33), its application in clinical practice remains limited in much of the world. Few countries have managed to integrate national teledentistry programmes, because this requires consolidation within health systems (34). In this regard, this study is a real pilot project within the public health system in rural areas.

Oral care should be comprehensive and ongoing, structured around three key areas: prevention, active treatment of conditions, and long-term preventive monitoring. This continuity of care is essential for optimising clinical outcomes and quality of life in relation to oral health. However, it is difficult to find units within the public system and primary care that offer comprehensive oral care programmes, probably due to their complexity, the burden of care or accessibility (35).

In this study, the majority of video consultations proved to be self-contained processes (i.e., they enabled the complete resolution of the case without the need for an in-person referral) and required less time to complete. These results could be explained, firstly, by the nature of the most common reasons for consultation, such as the delivery of diagnostic test results and reviews of prescribed treatments. Secondly, this greater time efficiency could be linked to the learning curve and specific training of healthcare professionals in the use of digital health tools. In this regard, our work once again highlights the need for specific and humanised training in VC (24, 25), coinciding with El Tantawi et al. (2023) (35), in which a Delphi study was conducted to develop a survey on the acceptance of teledentistry for dentists, showing in an initial phase the need for training, capacity building and investment in infrastructure from universities onwards. Along these lines, the SSPA (Andalusia) conducts annual accredited courses for healthcare professionals using high-fidelity clinical simulation methodology (25), following a guide for humanised action in video consultations (24).

The analysis of the digital divide in this study revealed a direct link to the technical difficulties reported. Specifically, patients aged 55 and over (Group 3) had the highest rate of connection issues during video consultations. This finding highlights the need to address technological barriers among adult patients to ensure equitable access to digital healthcare. This population, often less familiar with technology and with limited access to high-speed internet, faces significant challenges in establishing and maintaining a stable connection. However, the study also highlighted a positive finding: a considerable portion of these connectivity problems were resolved thanks to the presence of a caregiver or family member who assisted the patient. This assistance not only mitigated technical difficulties but also underscores the crucial role of social and family support in facilitating the adoption of telemedicine in communities facing a digital divide. This finding suggests that, in order to improve the accessibility of VC, it is essential to consider and integrate strategies that involve caregivers and family members in the care process, in addition to providing adequate technological training.

This opportunity for oral care and follow-up presents the main obstacle to implementing teledentistry, as both professionals and patients must learn a new telematic language and adapt to different forms of communication. Success depends on adequate training in video consultations, since without it, the alternative will not be successfully implemented and will generate rejection; dentists will fear not making a correct diagnosis or that the patient will not perceive the treatment as safe as in a face-to-face consultation, while patients may show technological fear or a feeling of abandonment, which was already experienced by some groups since the pandemic (24).

With regard to VC time by age group, there were no significant differences between groups, probably because G1 and G2 are patients familiar with the use of technology and G3 because most are patients accompanied by family members or carers of similar ages to those in group G2 (grandparents with children).

For our elderly, the pandemic further increased the difficulty of accessing healthcare, especially for those who are medically frail or institutionalised and whose fear of contagion, among other factors, prevented them from being treated in the conventional manner, unfortunately increasing their comorbidity (36). The need for comprehensive oral health care in primary care is of vital importance, and there is a clear need to implement strategies aimed at ensuring the quality of dental care for this group and thus promoting healthy ageing. In this regard, the results of this study show the possibility of monitoring these patients without the need for travel, and although one of the limitations observed in the study and in this age group (G3 > 55 years) has been access to these technologies, requiring the help of a family member or carer, it should be borne in mind that elderly patients often attend appointments accompanied by someone else, which implies greater logistical organisation for the family member/carer in terms of individual management and time and/or expense, such as taxis or vehicles to travel to the centre. In this regard, the family member has not had to travel, which could be a significant saving in time and money and could become an emerging opportunity for institutionalised elderly people who need to incorporate basic technological systems into their residences, or in home care settings, becoming a tool that reduces this physical barrier and promotes healthy ageing (14).

In terms of satisfaction, the resolution rate and time efficiency observed in our study are in line with the levels of satisfaction reported in the international literature on teledentistry. In the field of specialist dentistry, Parker and Chia (2021) (10, 11) demonstrated in their study of patient and professional satisfaction that over 90% of patients and 95% of clinicians would recommend video consultations, highlighting a preference of over 89% for the virtual format over face-to-face consultations for follow-up appointments and orthodontics. Similarly, in research on maxillofacial surgery, it has been demonstrated that there are no significant differences between the accuracy of in-person and telematic diagnoses, with a marked patient preference (up to 95%) for monitoring their condition via video consultation (20). These data suggest that, when the reason for the consultation is appropriate—such as treatment follow-up or the delivery of results, as observed in our rural sample—satisfaction is not only comparable to conventional care but is perceived as a preferred alternative due to its practicality.

When viewed within the context of rural settings, our findings align with those reported by Helvey et al. (2023) (37), who found that satisfaction levels with telemedicine in rural communities are even slightly higher than those in urban areas. This reinforces the argument that on-demand telemedicine can be a critical tool for reducing disparities in access to healthcare.

Finally, the positive impact on our group of people aged 55 and over, supported by the figure of the carer, fits into a global trend of success in virtual geriatric care. According to the systematic review by Şahin et al. (2024) (38), telemedicine for older adults has proven to be as effective as, or more effective than, standard care in terms of feasibility and the management of chronic conditions, with patients reporting that they are mostly satisfied or very satisfied. These findings validate our approach: far from being an emergency solution linked solely to pandemic restrictions, video consultations are establishing themselves as a sustainable and humanised strategy that reduces comorbidity and promotes healthy ageing, particularly when social support is integrated to bridge the digital divide.

### 4.1 Future directions

It is important to consider the patient experience. Understanding how patients interact with tele-dentistry services reveals implementation challenges and opportunities that may not be obvious from other perspectives.

Recent research is already exploring this aspect, identifying key factors that influence end-user acceptance of teledentistry. These findings suggest that, for the successful integration of teledentistry into dental curricula, a comprehensive approach is essential. This will improve the knowledge and experience of future dentists to meet the changing needs of their patients and adapt to evolving healthcare services.

While telemedicine offers numerous benefits, it also faces challenges in rural areas, such as limited connectivity, lack of infrastructure, and the need for training for professionals and patients. Overcoming these barriers requires investments in infrastructure, education campaigns, and collaboration with local communities.

### 4.2 Limitations and strengths

Although the pilot nature of the study limits the statistical power, particularly in subgroup analyses, and may increase the risk of type II errors, these results should be interpreted with caution. Additionally, the use of a convenience sample restricted to patients with access to digital devices introduces a potential selection bias, which may limit the generalizability of the findings and underestimate existing barriers to access in the broader rural population.

Furthermore, the effectiveness of video consultations is inherently dependent on the type of pathology addressed, as only those conditions considered clinically appropriate for remote management were included.

Despite these limitations, this study has several strengths. It represents a real-world pilot implementation, rather than a simulated scenario, integrated within the Andalusian Public Health System. The use of secure corporate infrastructure (Citrix, Diraya, Mercurio) ensured data protection and confidentiality in routine clinical practice.

In addition, the findings highlight the potential of video consultations to reduce geographical barriers in rural settings. The results highlight that the success of the study lies not only in the technology, but also in the specific training of healthcare professionals. Video consultations have been defined not merely as ‘standard video calls’, but as a structured clinical procedure, which makes this model scalable to other healthcare categories or specialities within the public primary care system. The role of caregivers emerged as a key facilitating factor, particularly among older patients with limited digital skills, partially mitigating the digital divide.

Overall, this study provides preliminary evidence supporting the feasibility and acceptability of video consultations in rural dental care, suggesting their potential to optimize follow-up processes while maintaining the quality of care. In short, the study validates humanized and accessible dental care in rural settings, optimizing clinical follow-up without compromising the integrity of the process.

## 5 Conclusions

Video consultations are an effective and viable tool in rural areas, especially for resolving issues such as communicating results or preparing for surgery. In particular, although many of those over 55 had connection problems, more than half of them were able to solve them with the help of a family member or carer, which underlines the importance of social support for the success of telemedicine in these areas, showing that, with the right support, video consultation is a valuable tool that can improve the accessibility and efficiency of primary care, especially in populations with greater technological challenges.

## Data Availability

The dataset generated and analyzed during the current study are not publicly avalaible due to privacy or ethical restrictions but are available from the corresponding author upon reasonable request.
